# X-Linked Retinoschisis Masquerading Uveitis

**DOI:** 10.3390/jcm12113729

**Published:** 2023-05-29

**Authors:** Luca Mautone, Johannes Birtel, Yevgeniya Atiskova, Vasyl Druchkiv, Nicole Stübiger, Martin S. Spitzer, Simon Dulz

**Affiliations:** Department of Ophthalmology, University Medical Center Hamburg-Eppendorf, Martinistr. 52, 20246 Hamburg, Germany

**Keywords:** X-linked retinoschisis, masquerade syndrome, uveitis, *RS1*, phenocopy, phenotyping

## Abstract

X-linked retinoschisis (XLRS) shows features also seen in patients with uveitis and is recognized as an uveitis masquerade syndrome. This retrospective study aimed to describe characteristics of XLRS patients with an initial uveitis diagnosis and to contrast these to patients with an initial XLRS diagnosis. Patients referred to a uveitis clinic, which turned out to have XLRS (*n* = 4), and patients referred to a clinic for inherited retinal diseases (*n* = 18) were included. All patients underwent comprehensive ophthalmic examinations, including retinal imaging with fundus photography, ultra-widefield fundus imaging, and optical coherence tomography (OCT). In patients with an initial diagnosis of uveitis, a macular cystoid schisis was always interpreted as an inflammatory macular edema; vitreous hemorrhages were commonly interpreted as intraocular inflammation. Patients with an initial diagnosis of XLRS rarely (2/18; *p* = 0.02) showed vitreous hemorrhages. No additional demographic, anamnestic, and anatomical differences were found. An increased awareness of XLRS as a uveitis masquerade syndrome may facilitate early diagnosis and may prevent unnecessary therapies.

## 1. Introduction

Uveitis masquerade syndromes (UMSs) describe non-inflammatory conditions characterized by intraocular cells which are not secondary to an immune-mediated or infectious process, but which are interpreted as inflammatory [1,2]. The term masquerade syndrome was introduced by Theodore, reporting a conjunctival carcinoma presenting as chronic conjunctivitis [3]. The most prominent UMSs are neoplastic pathologies, including vitreoretinal/intraocular lymphoma in adults and retinoblastoma in children [2,4]. However, several non-neoplastic diseases may also masquerade as uveitis, including inherited retinal diseases and X-linked retinoschisis (XLRS) [5,6,7].

XLRS is caused by variants in the retinoschisin 1 (*RS1*) gene, the gene product is important for the maintenance of the retinal structure [8]. Clinical characteristics of XLRS include macular cystoid schisis, a macular spoke wheel pattern, peripheral retinoschisis, recurrent vitreous hemorrhages, and retinal vessel abnormalities such as vascular attenuation or shealting [9,10,11,12]. A precise diagnosis appears decisive for XLRS patients, enabling timely patient/family counseling, and may enable inclusion in upcoming clinical trials [13,14,15,16]. Furthermore, an early diagnosis and differentiation from uveitis may avoid initiation of local or systemic immunosuppression with potential side effects and ineffective outcomes. This may even have an impact on family planning, due to the known mutagenic potential of several immunosuppressive agents used for the treatment of uveitis [17].

This study aims to characterize XLRS patients initially diagnosed with uveitis and to contrast these with patients with an initial XLRS diagnosis.

## 2. Materials and Methods

This retrospective study was conducted at the Department of Ophthalmology, University Medical Center, Hamburg-Eppendorf, Germany, and adhered to the tenets of the Declaration of Helsinki. Due to the retrospective and anonymized data collection and analysis, no ethical approval was required.

Inclusion criteria were (1) the clinical diagnosis of XLRS and (2) a mutation in *RS1* (*n* = 17) or a pedigree indicating X-linked inheritance when molecular screening was not performed. Subjects reviewed between 1 January 2012 and 1 March 2022 were included. Genetic testing, as part of the routine diagnostic workup, was performed as described previously [18]. Patients were divided into two groups: patients referred with a suspected diagnosis of uveitis [uXLRS], and patients referred to a clinic for inherited retinal disease [pXLRS]. All patients had been previously assessed/treated by local ophthalmologists.

Each subject underwent a comprehensive ophthalmic examination, including best corrected visual acuity (BCVA, logMAR) testing, applanation tonometry, slit lamp biomicroscopy, and dilated fundoscopy. Multimodal retinal imaging included fundus photography (Topcon, Tokyo, Japan), ultra-widefield fundus autofluorescence and digital imaging (Optos, Dunfermline, UK), swept-source optical coherence tomography (SS-OCT; Topcon, Tokyo, Japan), and/or spectral domain OCT (SD-OCT, Heidelberg Engineering, Heidelberg, Germany), as previously described [13,14].

Family history, onset of visual symptoms, history of intraocular surgeries, and phenotypic characteristics, including macular cystoid schisis, macular spoke wheel patterns, peripheral retinoschisis, vitreous veil, vascular sheeting, falciform fold, white spiculations, schisis foramen, and vitreous hemorrhage were assessed. OCT imaging was used for central retinal thickness measurements, to evaluate the presence of macular cystoid schisis, outer retinal atrophy, epiretinal membranes, and subretinal fluid accumulation. The ETDRS grid was used to assess the central retinal thickness within the central one-millimeter diameter centered on the fovea. The grid alignment and OCT segmentation were corrected manually if needed.

Statistical analyses were performed with R Core Team 2021 (Vienna, Austria) [19]. Statistical differences between the two groups were analyzed via the independent *t*-Test, Mann–Whitney Test and Fisher’s Exact Test.

## 3. Results

Twenty-seven patients with a diagnosis of XLRS were reviewed. Five subjects were excluded, due to a lack of genetic testing and a pedigree not indicating X-linked inheritance. Clinical data are summarized in Table 1.

**Figure 1:**
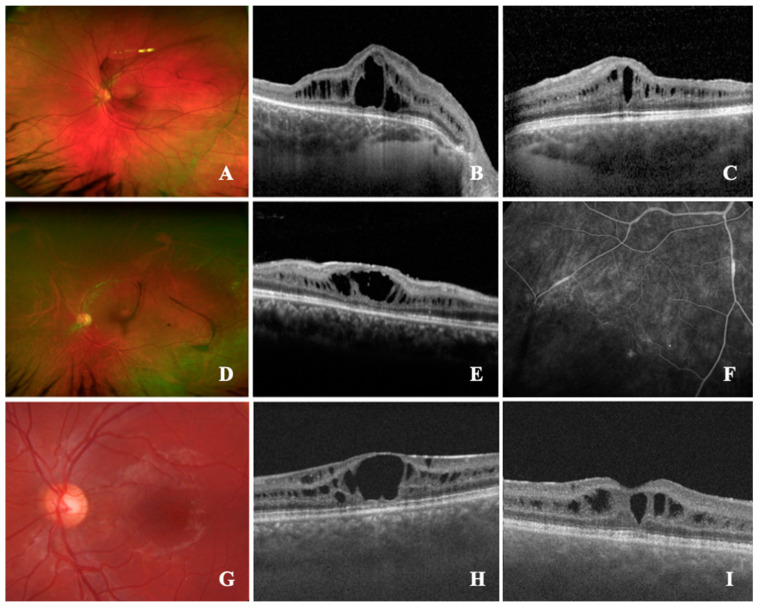
**Retinal findings of X-linked retinoschisis (XLRS) patients referred for an uveitis assessment**. (**A**–**C**): #19; (**A**), ultra-widefield fundus image (UWF) demonstrates the presence of retinal metallic sheen (MS) and vitreous veils; (**B**,**C**), spectral-domain optical coherence tomography (SD-OCT) shows macular cystoid schisis (MCS) of the inner nuclear layer (INL) and outer plexiform layer (OPL). (**D**–**F**): #20; (**D**), UWF showing vascular sheathing, white spiculations, and peripheral retinoschisis; (**E**), SD-OCT shows MCS of INL; (**F**), late venous fluorescein angiography showing peripheral retinal vascular defects with leakages and nonperfusion areas. (**G**,**H**): #21; G, color fundus photography revealing spoke wheel pattern configuration of the macula; (**H**), SD-OCT delineating the MCS in ganglion cell layer (GCL), INL, OPL and outer nuclear layer (ONL). (**I**): #22; SD-OCT with MCS of GCL and INL.

Four out of twenty-two (18%) patients were referred with the diagnosis of uveitis: one (#19) with posterior uveitis with macular edema, one (#20) with posterior uveitis with ME and retinal vasculitis, and two (#21 and #22) with intermediate uveitis with macular edema. In all, an underlying systemic rheumatological, immunological, and infectious disease was excluded. The history of these four patients is outlined below:

**Case 1 (#19):** An asymptomatic 14-year-old male was referred for the treatment of a bilateral posterior uveitis with macular edema, and for the investigation of its underlying systemic disease. BCVA was 1.0 (right eye, OD) and 0.2 (left eye, OS) logMAR, respectively; the right eye had a known history of amblyopia due to posterior subcapsular cataract, and the patient underwent cataract surgery at the age of four. Clinical examination showed normal anterior segments. On fundus examination, bilateral active vitreous bleeding and retinal hammered metal appearance were seen (Figure 1A). On the left eye, peripheral retinoschisis and vitreous veil had occurred. Retinal imaging (Figure 1B,C) demonstrated a macular cystoid schisis of the inner nuclear layer (INL) and outer plexiform layer (OPL), peripheral retinoschisis, but no macular spoke wheel pattern. Comprehensive patient history revealed that a maternal cousin had a known history of XLRS which was also confirmed in this patient (*RS1*, c.626G > A).

**Case 2** (#**20)**: A 37-year-old male was referred for a bilateral posterior uveitis with vasculitis and macular edema. BCVA was 1.3 (OD) and 0.2 (OS) logMAR, respectively. The right eye had a known history of amblyopia and the patient underwent vitrectomy at the age of one, due to suspect toxoplasmosis. Clinical examination showed posterior cataract (OD) peripheral retinoschisis, and vascular sheathing, and white spiculations were seen in both eyes (Figure 1D). OCT imaging demonstrated a macular atrophy with parafoveal cystoid spaces in the INL (OD) and macular cystoid schisis of the INL (OS, Figure 1E). Fluoresceine angiography showed unspecific vascular changes of the periphery with leakage and peripheral nonperfusion (Figure 1F,G), previously interpreted as peripheral occlusive vasculitis. Due to these characteristics and a macular spoke wheel pattern, as well as blood cells in the vitreous of the left eye, genetic testing was initiated, confirming XLRS (*RS1*, c.325g > C).

**Case 3 (#21):** An asymptomatic 19-year-old male was referred for a second opinion of intermediate uveitis with a persistent macular edema and intraocular inflammation, despite a systemic treatment with 7.5 mg peroral prednisone once a day. Previously, a therapy with peroral acetazolamide (250 mg twice daily) and prednisolone (initially 75 mg daily, tapered over 3 months) was initiated. Due to elevated intraocular pressure, following the systemic corticosteroid therapy, timolol (0.5%) eyedrops were applied twice daily. BCVA was 0.4 logMAR (both eyes, OU). Clinical examination showed regular anterior segments and erythrocytes in the vitreous body. Fluoresceine angiography demonstrated no vascular abnormalities; on OCT imaging, bilateral macular cystoid schisis was present in the ganglion cell layer, INL, OPL, and outer nuclear layer (ONL), and a macular spoke wheel pattern was visible (Figure 1H,I). These alterations pointed to XLRS, which was confirmed by molecular testing (*RS1*, c.460c > T). Subsequently, the topical and systemic medication was terminated.

**Case 4 (#22):** An asymptomatic 19-year-old male was urgently referred for the treatment of intermediate uveitis with macular edema. The family history for eye diseases was unremarkable. BCVA was 0.2 logMAR (OU). Clinical examination demonstrated a clear lens and a macular spoke wheel pattern, as well as a metallic sheen of the retina. No active vitreous hemorrhage was seen. On OCT imaging, macular cystoid schisis of the INL and ONL was seen. Genetic testing confirmed a mutation in the *RS1* gene. The patient asked for an off-label therapy with topical dorzolamide, which was initiated.

Comparing the uXLRS and pXLRS patients (Table 2), no age difference at initial presentation was seen (mean age uXLRS: 22.25, SD ± 10.11 years; mean age pXLRS: 24.89, SD ± 13.80 years; *p* = 0.72). No difference in the onset of symptoms was found between groups, only 1 case in the pXLRS group (#12) reported acute symptoms due to vitreous hemorrhage, while all other patients complained of chronic visual symptoms. Furthermore, no difference regarding an XLRS family history, history of cataract surgery, or vitreoretinal surgeries was seen. At presentation, the uXLRS patients showed a better mean BCVA of the better eye (0.28, SD ± 0.1 logMAR) than the pXLRS patients (24.89, SD ± 13.80 logMAR, *p* = 0.03). The mean BCVA of the worse eye did not differ between groups.

No differences regarding the morphological retinal findings except the presence of vitreous hemorrhage were found. An active vitreous hemorrhage occurred in three (75%) patients in the uXLRS-group and two (11%) patients in the pXLRS -group, respectively (*p* = 0.02). In all four uXLRS patients, macular cystoid schisis was initially interpreted as uveitis-associated macular edema. On OCT imaging, no structural differences between the two groups, regarding the central retinal thickness, macular atrophy, presence of ERM, or macular cystoid schisis were identified (Table 2).

## 4. Discussion

XLRS represents a uveitis masquerade syndrome—the distinction between this inherited retinal disease and uveitis appears to be fundamental [6,7]. In this cohort, 4 out of 22 XLRS patients were primary diagnosed as uveitis, which is in accordance with a study by Kousal et al., reporting 3 out of 21 XLRS patients initially diagnosed as intermediate uveitis [6]. De Carvalho Mendes Paiva et al. reported an 8-month-old male initially treated for presumptive ocular toxoplasmosis, due to the loss of red reflex, vitreous opacity, and thickened posterior hyaloid, who later turned out to be an XLRS patient [7]. In this case, an XLRS subject received a systemic medication, due to the initial diagnosis of intermediate uveitis with macular edema. This underlines the risk of the administration of treatment in XLRS patients if they are diagnosed with uveitis masquerade syndrome.

This study investigated the differences between patients with an initial diagnosis of XLRS and uveitis. No difference in demographics and onset of visual symptoms was seen. The positive family history for XLRS—which is suggestive for this retinal disease, due its x-linked recessive inheritance—also did not differ between groups. Several morphological features of XLRS may contribute to a misleading diagnosis: a mild vitreous hemorrhage may be interpreted as intravitreal inflammation, a macular cystoid schisis may mimic inflammatory macular edema and the peripheral retinal vessel abnormalities as vascular attenuation, or sheathing may mimic retinal vasculitis [11]. We report a higher prevalence of vitreous hemorrhages in patients initially diagnosed as uveitis compared to patients with a primary XLRS diagnosis. No further differences in retinal morphological characteristics, such as vitreous veils, metallic sheen, peripheral retinoschisis, macular spoke wheel pattern, falciform folds, schisis foramen, and white spiculations were revealed. Additionally, structural examinations with OCT imaging showed no differences regarding the presence of macular cystoid schisis, epiretinal membranes, and outer retinal atrophy. Macular cystoid schisis is a common feature in XLRS (70.4–88% of cases), and it may involve the macula only or extend throughout the macula, outside the temporal arcades and nasal to the optic disc [9,10,20,21,22]. Especially if the schisis is limited to the macula, the differentiation from an inflammatory macular edema—present in 35.3% of intermediate and 18.9% of posterior uveitis—may be challenging [21,23]. Even vascular changes may mimic intraocular inflammation in XLRS. As seen in one case within this study, fluorescein angiography may reveal extra-macular retinal vascular leakage and peripheral nonperfusion, even in the absence of peripheral retinoschisis [4,24,25]. Furthermore, in the absence of retinal XLRS, finding a macular spoke wheel pattern (49.3% and 61.1–70% of cases, respectively) may misguide to an uveitis diagnosis [10,20]. However, the peripheral retinoschisis is not specific to XLRS, as it could occur in 95.6% of eyes with intermediate uveitis [26].

How can an ophthalmologist distinguish XLRS from uveitis? A positive family history, an X-linked inheritance trait, bilateral disease, and/or disease onset at a young age may be a first hint. Furthermore, retinal imaging may support the diagnostic work-up. OCT imaging, for instance, may assist in distinguishing between macular cystoid schisis and an inflammatory macular edema. Fluoresceine angiography may assist, as it reveals petaloid defects with no leakage in XLRS, whereas a macular edema with leakage is commonly seen in uveitis [4,24,27,28]. Indocyanine green angiography may display a limited hyperfluorescence starfish-like pattern of the macula—corresponding to the cystoid cavities—and hypofluorescence radial lines without peripheral defects in XLRS [29]. In patients with uveitis, indocyanine green angiography often shows different patterns which may be beneficial for differential diagnosis [30]. Lastly, electroretinography might also be helpful, as XLRS may be associated with an electronegative b-wave [9,20].

In conclusion, XLRS may masquerade as uveitis. Vitreous hemorrhaging with concomitant macular cystoid schisis and other morphological findings may mimic intraocular inflammatory activity and may mislead to an uveitis diagnosis. Genetic testing should be performed in suspicious cases.

## Figures and Tables

**Table 1:** Clinical characteristics. BCVA: Best-Corrected Visual Acuity. OD: Right Eye. OS: Left Eye. AT: Atrophy. ERM: Epiretinal Membrane. MCS: Macular Cystoid Schisis. MS: Metallic Sheen. OCT: Optical Coherence Tomography. PS: Peripheral Schisis. SF: Schisis Foramen. SWP: Spoke Wheel Pattern. VH: Vitreous Hemorrhage. VS: Vascular Sheathing. VV: Vitreous Veil. WS: White Spiculations. XLRS: X-Linked Retinoschisis.

Patient (#)	Age	BCVA (OD/OS) [LogMAR]	Pseudophakia	History of Vitreoretinal Surgery	Retinal Characteristics	OCT Characteristics
**Patients with an initial diagnosis of XLRS (pXLRS)**						
1	25	0.5/0.6	No	Yes	SWP, PS, VV, WS, MS, SF	MCS
2	52	0.3/1.0	No	Yes	SWP, MS	ERM, MCS
3	5	0.4/0.6	No	No	SWP, PS, VV, WS, MS, SF	MCS
4	45	1.0/0.8	No	Yes	MS	MCS, AT
5	39	1.5/0.6	Yes	Yes	PS, VS, VV, VH, MS, SF	MCS, AT
6	33	0.5/1.0	No	No	PS, VV, MS, SF	ERM, MCS
7	20	0.3/0.2	No	No	SWP, PS, MS	MCS
8	11	1.0/1.0	No	No	SWP, VS, MS	MCS
9	5	0.4/1.3	No	No	PS, VS, VV, VH, WS, MS, SF	MCS
10	28	0.7/0.6	No	Yes	PS, VS, VV, MS, SF	ERM, MCS
11	25	0.7/0.7	No	No	SWP, MS	MCS, AT
12	8	0.6/0.2	No	No	SWP, MS	MCS
13	25	1.0/1.0	No	No	SPW	MCS
14	28	1.4/0.7	No	No	PS	ERM, MCS, AT
15	26	1.0/1.0	No	No	SWP, PS, VV, WS, SF	MCS
16	31	0.6/1.1	No	No	SWP, PS, VV, FF, MS, SF	ERM, MCS
17	36	1.0/1.0	No	No	PS	AT
18	6	0.5/0.3	Yes	Yes	SWP, PS, VV	MCS
**Patients with an initial diagnosis of uveitis (uXLRS)**						
19	14	1.0/0.2	Yes	No	PS, VV, VH, WS, MS	MCS
20	37	1.3/0.3	No	Yes	SPW, PS, VS, VV, VH, WS, MS	ERM, MCS, AT
21	19	0.4/0.4	No	No	SWP, VH	MCS
22	19	0.2/0.2	No	No	SWP, MS	MCS

**Table 2:** Patient characteristics. Characteristics of patients referred with a suspected diagnosis of uveitis [uXLRS], and patients referred with a suspected inherited retinal disease [pXLRS]. BCVA: Best Corrected Visual Acuity. CRT: Central Retinal Thickness. ERM: Epiretinal Membrane. OCT: Optical Coherence Tomography. SD: Standard Deviation. XLRS: X-Linked Retinoschisis.

	uXLRS-Group (*n* = 4)	pXLRS-Group (*n* = 18)	Total (*n* = 22)
**Age (years)**			
mean (SD)	22.25 (10.11)	24.89 (13.80)	24.41 (13.03)
median (range)	19 (14–37)	25 (5–52)	25 (5–52)
**Family history of XLRS**	1 (25.0%)	9 (50.0%)	10 (25.5%)
**Mean BCVA (LogMAR)**			
better eye (SD)	0.28 (0.10)	0.60 (0.28)	0.54 (0.28)
worse eye (SD)	0.73 (0.51)	0.96 (0.41)	0.92 (0.43)
**Pseudophakia**	1 (25.0%)	2 (11.1%)	3 (13.6%)
**History of retinal surgeries**	1 (25.0%)	6 (33.3%)	7 (31.8%)
**Spoke wheel pattern**	3 (75.0%)	11 (61.1%)	13 (63.6%)
**Peripheral schisis**	2 (50.0%)	12 (66.7%)	14 (63.6%)
**Metallic sheen**	3 (75.0%)	13 (72.2%)	16 (72.7%)
**Vascular sheathing**	1 (25.0%)	4 (22.2%)	5 (22.7%)
**Vitreos veil**	2 (50.0%)	9 (50.0%)	11 (5.0%)
**Falciform folds**	0	1 (5.5%)	1 (4.5%)
**White spiculations**	1 (25.0%)	4 (22.2%)	5 (22.7%)
**Schisis foramen**	0	8 (44.4%)	8 (36.4%)
**Vitreous hemorrage**	3 (75.0%)	2 (11.1%)	5 (22.7%)
**OCT: maculoschisis**	4 (100.0%)	17 (94.4%)	21 (95.5%)
**OCT: macular atrophy**	1 (25.0%)	5 (27.8%)	6 (27.2%)
**OCT: ERM**	1 (25.0%)	5 (27.8%)	6 (27.2%)
**CRT (µm)**			
mean (SD)	387.62 (62.86)	406.53 (153.92)	402.93 (140.71)

## Data Availability

Data that support the findings of this study are included in the manuscript.

## References

[1] Grange L.K., Kouchouk A., Dalal M.D., Vitale S., Nussenblatt R.B., Chan C.C., Sen H.N. (2014). Neoplastic Masquerade Syndromes in Patients with Uveitis. Am. J. Ophthalmol..

[2] Rothova A., Ooijman F., Kerkhoff F., Van der Lelij A., Lokhorst H.M. (2001). Uveitis Masquerade Syndromes. Ophthalmology.

[3] Theodore F.H. (1967). Conjunctival Carcinoma Masquerading as Chronic Conjunctivitis. Eye. Ear. Nose Throat Mon..

[4] Rao P., Robinson J., Yonekawa Y., Thomas B.J., Drenser K.A., Trese M.T., Capone A. (2016). Wide-Field Imaging of Nonexudative and Exudative Congenital X-Linked Retinoschisis. Retina.

[5] Rothova A., Groen F., Ten Berge J.C.E.M., Lubbers S.M., Vingerling J.R., Thiadens A.A.H.J. (2021). Causes and Clinical Manifestations of Masquerade Syndromes in Intraocular Inflammatory Diseases. Retina.

[6] Kousal B., Hlavata L., Vlaskova H., Dvorakova L., Brichova M., Dubska Z., Langrova H., Vincent A.L., Dudakova L., Liskova P. (2021). Clinical and Genetic Study of X-Linked Juvenile Retinoschisis in the Czech Population. Genes.

[7] Paiva A.d.C.M., Teixeira F.H.F., Carvalho E.M., Santos N.S., Biancardi A.L., Curi A.L.L. (2022). An Atypical Early-Onset X-Linked Retinoschisis Mimicking Uveitis Masquerade Syndrome. Arq. Bras. Oftalmol..

[8] Weber B.H.F., Schrewe H., Molday L.L., Gehrig A., White K.L., Seeliger M.W., Jaissle G.B., Friedburg C., Tamm E., Molday R.S. (2002). Inactivation of the Murine X-Linked Juvenile Retinoschisis Gene, Rs1h, Suggests a Role of Retinoschisin in Retinal Cell Layer Organization and Synaptic Structure. Proc. Natl. Acad. Sci. USA.

[9] Hahn L.C., van Schooneveld M.J., Wesseling N.L., Florijn R.J., ten Brink J.B., Lissenberg-Witte B.I., Strubbe I., Meester-Smoor M.A., Thiadens A.A., Diederen R.M. (2022). X-Linked Retinoschisis: Novel Clinical Observations and Genetic Spectrum in 340 Patients. Ophthalmology.

[10] Georgiou M., Finocchio L., Fujinami K., Fujinami-Yokokawa Y., Virgili G., Mahroo O.A., Webster A.R., Michaelides M. (2022). X-Linked Retinoschisis: Deep Phenotyping and Genetic Characterization. Ophthalmology.

[11] George N.D., Yates J.R., Moore A.T. (1996). Clinical Features in Affected Males with X-Linked Retinoschisis. Arch. Ophthalmol..

[12] Molday R.S., Kellner U., Weber B.H.F. (2012). X-Linked Juvenile Retinoschisis: Clinical Diagnosis, Genetic Analysis, and Molecular Mechanisms. Prog. Retin. Eye Res..

[13] Birtel J., Gliem M., Holz F.G., Herrmann P. (2018). Imaging and Molecular Genetic Diagnostics for the Characterization of Retinal Dystrophies. Ophthalmologe.

[14] Birtel J., Yusuf I.H., Priglinger C., Rudolph G., Charbel Issa P. (2021). Diagnosis of Inherited Retinal Diseases. Klin. Monbl. Augenheilkd..

[15] Cukras C.A., Huryn L.A., Jeffrey B.P., Turriff A., Sieving P.A. (2018). Analysis of Anatomic and Functional Measures in X-Linked Retinoschisis. Investig. Ophthalmol. Vis. Sci..

[16] Pennesi M.E., Yang P., Birch D.G., Weng C.Y., Moore A.T., Iannaccone A., Comander J.I., Jayasundera T., Chulay J. (2022). XLRS-001 Study Group Intravitreal Delivery of RAAV2tYF-CB-HRS1 Vector for Gene Augmentation Therapy in X-Linked Retinoschisis—1 Year Clinical Results. Ophthalmol. Retin..

[17] Leroy C., Rigot J.-M., Leroy M., Decanter C., Le Mapihan K., Parent A.-S., Le Guillou A.-C., Yakoub-Agha I., Dharancy S., Noel C. (2015). Immunosuppressive Drugs and Fertility. Orphanet J. Rare Dis..

[18] Birtel J., Eisenberger T., Gliem M., Müller P.L., Herrmann P., Betz C., Zahnleiter D., Neuhaus C., Lenzner S., Holz F.G. (2018). Clinical and Genetic Characteristics of 251 Consecutive Patients with Macular and Cone/Cone-Rod Dystrophy. Sci. Rep..

[19] R Development Core Team (2012). A Language and Environment for Statistical Computing, Version 4.1.2 (2021-11-01).

[20] Orès R., Mohand-Said S., Dhaenens C.-M., Antonio A., Zeitz C., Augstburger E., Andrieu C., Sahel J.-A., Audo I. (2018). Phenotypic Characteristics of a French Cohort of Patients with X-Linked Retinoschisis. Ophthalmology.

[21] Gregori N.Z., Lam B.L., Gregori G., Ranganathan S., Stone E.M., Morante A., Abukhalil F., Aroucha P.R. (2013). Wide-Field Spectral-Domain Optical Coherence Tomography in Patients and Carriers of X-Linked Retinoschisis. Ophthalmology.

[22] Han I.C., Whitmore S.S., Critser D.B., Lee S.Y., DeLuca A.P., Daggett H.T., Affatigato L.M., Mullins R.F., Tucker B.A., Drack A.V. (2019). Wide-Field Swept-Source OCT and Angiography in X-Linked Retinoschisis. Ophthalmol. Retin..

[23] Levin M.H., Pistilli M., Daniel E., Gangaputra S.S., Nussenblatt R.B., Rosenbaum J.T., Suhler E.B., Thorne J.E., Foster C.S., Jabs D.A. (2014). Incidence of Visual Improvement in Uveitis Cases with Visual Impairment Caused by Macular Edema. Ophthalmology.

[24] Green J.L., Jampol L.M. (1979). Vascular Opacification and Leakage in X-Linked (Juvenile) Retinoschisis. Br. J. Ophthalmol..

[25] Odland M. (1981). Congenital Retinoschisis. Acta Ophthalmol..

[26] Pichi F., Srivastava S.K., Nucci P., Baynes K., Neri P., Lowder C.Y. (2017). Peripheral Retinoschisis in Intermediate Uveitis. Retina.

[27] Tugal-Tutkun I., Herbort C.P., Khairallah M. (2010). Angiography Scoring for Uveitis Working Group (ASUWOG) Scoring of Dual Fluorescein and ICG Inflammatory Angiographic Signs for the Grading of Posterior Segment Inflammation (Dual Fluorescein and ICG Angiographic Scoring System for Uveitis). Int. Ophthalmol..

[28] Ciardella A.P., Prall F.R., Borodoker N., Cunningham E.T. (2004). Imaging Techniques for Posterior Uveitis. Curr. Opin. Ophthalmol..

[29] Souied E.H., Goritsa A., Querques G., Coscas G., Soubrane G. (2005). Indocyanine Green Angiography of Juvenile X-Linked Retinoschisis. Am. J. Ophthalmol..

[30] Herbort C.P., LeHoang P., Guex-Crosier Y. (1998). Schematic Interpretation of Indocyanine Green Angiography in Posterior Uveitis Using a Standard Angiographic Protocol. Ophthalmology.

